# Delayed, recurrent bile leak from isolated right posterior sectoral duct injury after laparoscopic cholecystectomy: An unusual presentation

**DOI:** 10.1002/ccr3.6032

**Published:** 2022-07-18

**Authors:** Amit Sharma, Brianna Ruch, Yahya Alwatari, Sonia Lele, Doumit S. Bouhaidar

**Affiliations:** ^1^ Hume‐Lee Transplant Center Virginia Commonwealth University Richmond Virginia USA; ^2^ Division of Gastroenterology Virginia Commonwealth University Richmond Virginia USA

**Keywords:** bile leak, laparoscopic cholecystectomy, sectoral duct

## Abstract

Bile leak after cholecystectomy is associated with significant comorbidity. Biliary duct variant anatomy can complicate identification and management. We report a very rare presentation of recurrent delayed bile leaks years after laparoscopic cholecystectomy secondary to missed right posterior sectoral bile duct injury. Surgical intervention was required after the failure of conservative management.

## Graphical abstract

Right posterior sectoral duct injury should be considered in patients presenting with a delayed recurrent bile leak. Deep dissection into liver parenchyma during gallbladder fossa dissection should be avoided to prevent the occurrence of such injuries.

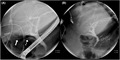

## 1 INTRODUCTION

Clinically significant bile leaks complicate about 0.3%–2.7% of cholecystectomies.^1^ Biliary tree anomalies may be present in up to 25% of the patient population, and aberrant right hepatic ducts are the most common.^2^ While a majority of bile leaks originate from the cystic duct (CD) stump or the subvesical duct of Luschka,^1^ those from aberrant sectoral bile ducts are rarely discussed.

Aberrant sectoral duct arises most commonly from the right liver segments and drain into the common hepatic duct (CHD) or CD. These ducts can represent the only route of biliary drainage for the portion of the right hepatic lobe they drain. From a surgical standpoint, the most clinically relevant sectoral variant to consider is when the CD runs alongside a low‐lying aberrant right sectoral duct, most commonly the right posterior sectoral duct (RPSD), which drains segments 6 and 7. It is present in 4.8%–8.4% of the population.^2^ Injuries to these ducts are likely underreported since they may be asymptomatic and often unrecognized as the injured area atrophies over time.

We present an unusual case of right posterior sectoral bile duct injury that presented as a delayed, recurrent bile leak, two and 7 years after laparoscopic cholecystectomy discussing challenges with diagnosis and management.

## 2 CASE REPORT

A 62‐year‐old female patient was referred to our surgical clinic for the evaluation of recurrent bile leak following an uneventful laparoscopic cholecystectomy 7 years ago, at an outside hospital. The patient had remained asymptomatic for about two years post‐cholecystectomy when she developed right upper quadrant abdominal pain. A perihepatic fluid collection that was percutaneously drained and was consistent with a bile leak. She then underwent an endoscopic retrograde cholangiopancreatography (ERCP) with sphincterotomy and biliary stent placement. Additionally, percutaneous transhepatic biliary drainage was performed due to the persistence of bile leak in the external drain (images from outside hospital were not available). After several weeks, she had eventual resolution of the bile leak followed by the removal of all the drains and stent. Patient remained asymptomatic for another 5 years before presenting to our gastroenterology department with a recurrent bile leak for which a percutaneous drain had been placed.

The patient presenting symptoms included sharp right upper quadrant abdominal pain radiating to right lower quadrant and shoulders. Associated symptoms included abdominal distension, diarrhea, nausea, and low‐grade fever. At initial presentation, liver function tests showed an AST of 269 units/L, ALT of 210 units/L, alkaline phosphatase of 146 units/L, and bilirubin (total) of 1.5 mg/dl (conjugated of 0.7 mg/dl). CBC demonstrated an initial leukocytosis (14.1 10e9/L). The WBC count was normal (9.7 10e9/L) on repeat draw 24 h. The patient treatment included prophylactic antibiotics course.

**FIGURE 1:**
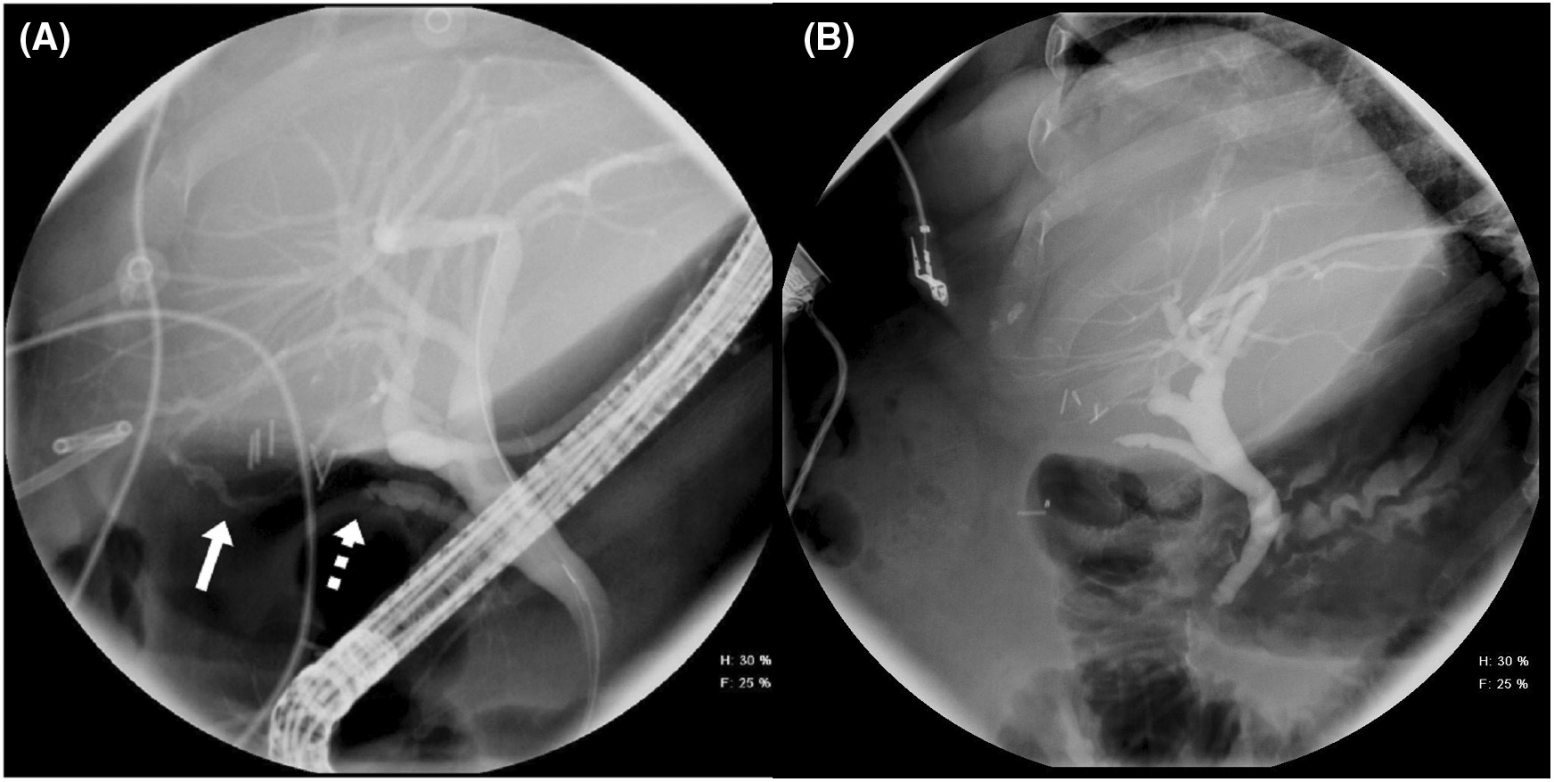
(A) Preoperative ERCP with irregular contrast filling near the gallbladder fossa consistent with bile leak from a right posterior sectoral duct branch (solid white arrow). Cystic duct remnant (dashed arrow) is seen without any evidence of leak. (B) Postoperative ERCP after removal of biliary stent showing no evidence of bile leak in the gallbladder fossa

**FIGURE 2:**
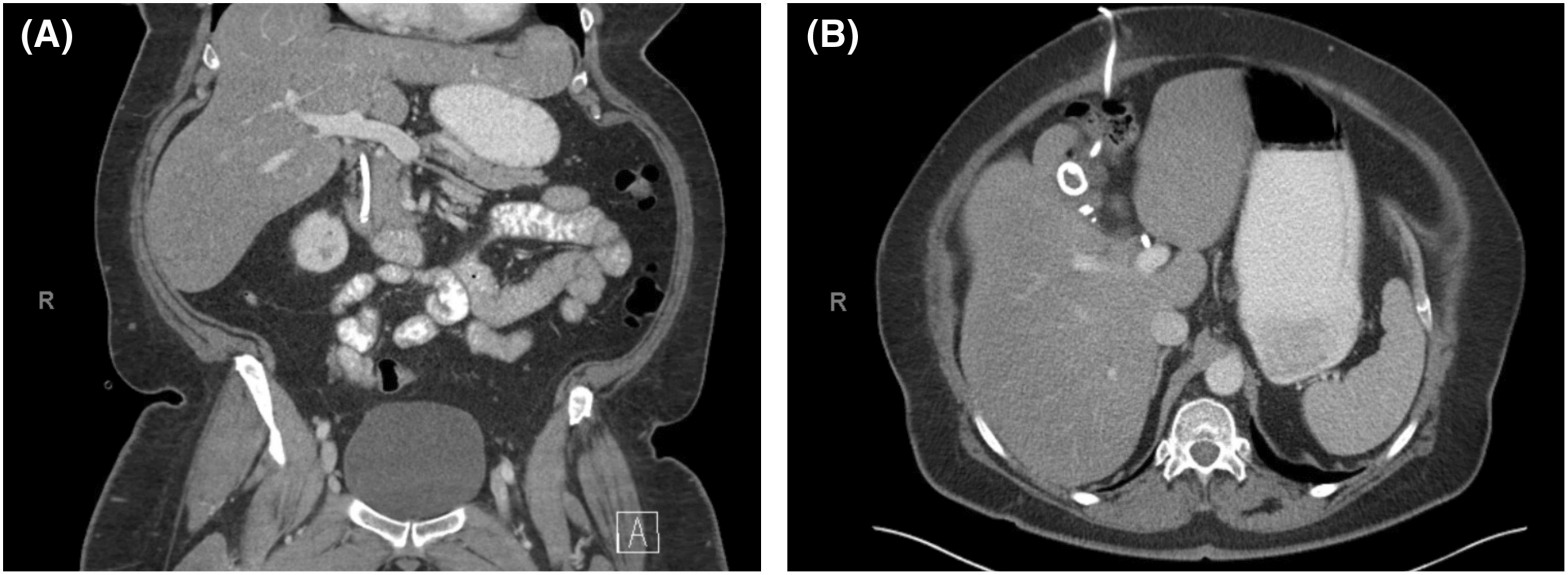
Preoperative abdominal CT demonstrating biliary stent in common bile duct (A). A right upper quadrant abdominal drainage catheter is in place and terminates in the gallbladder fossa (B)

She underwent an initial ERCP, which showed irregular contrast filling in the gallbladder fossa consistent with leak possibly from an isolated right posterior sectoral duct (Figure 1A). An internal biliary stent was placed to create an alternative path for bile flow and promote spontaneous closure of the leak. A repeat ERCP 2 months later showed possible leakage from the right posterior sectoral duct, and the patient continued to have 20–40 ml/d of bilious output in her percutaneous drain, indicating a failure of conservative management. An abdominal CT performed at this time demonstrated an appropriately placed biliary stent traversing the common bile duct, and a right upper quadrant drainage catheter terminating in the gallbladder fossa (Figure 2A,B). Amorphous complex fluid attenuation was also visible in the gallbladder fossa, and a continued leak was suspected. The patient was consented for an exploratory laparotomy for possible isolation and ligation or reconstruction of the leaking bile duct versus a partial wedge hepatic resection if the leaking duct was not identified.

Exploration was performed via a right subcostal incision. The percutaneous catheter was tracked into a chronic abscess cavity along the inferior edge of the liver and overlying the gallbladder fossa. The cavity was de‐roofed, and about 10 ml of bilio‐purulent fluid was drained. The common bile duct was identified. After debridement of the abscess cavity and irrigation with copious amounts of neomycin/bacitracin antibiotic solution, a pinpoint (3 mm diameter) area of bile leak was identified in the gallbladder fossa. This was probed with coronary dilators and confirmed to be the offending bile duct branch. The overlying scar tissue was excised, and the freshened edges of the duct were oversewn using multiple interrupted 5–0 polydioxanone sutures. The percutaneous drain was removed and a 19‐Fr surgical drain placed.

The patient had an uncomplicated recovery. The surgical drain was removed on postoperative Day 10 in the clinic. An ERCP was performed 6 weeks after surgery, and the biliary stent was removed (Figure 1B). 24 months after surgery, the patient continues to do well without any evidence of bile leak (serum total bilirubin of 0.6 mg/dl and serum ALP 100 units/L).

## 3 DISCUSSION

We report a very rare presentation of recurrent bile leak after cholecystectomy in context of aberrant anatomy that failed conservative management and required surgical intervention with good patient outcome. The exact etiology of this delayed bile leak is unclear; however, we postulate that it may be secondary to a likely tangential thermal injury to a superficial right posterior sectoral duct during dissection at the gallbladder fossa during index laparoscopic cholecystectomy.

Timing of presentation of bile ducts injury after cholecystectomy can be variable. If the duct injury is not recognized intraoperatively, postoperative bile leaks result in patients' reported symptoms of abdominal pain, nausea, loss of appetite, and lethargy. Posterior sectoral duct injuries may escape detection and opacification on intraoperative cholangiogram or postoperative ERCP due to the lack of communication of the injured ducts with the main biliary channels, thereby rendering angiographic assessment challenging. Diagnosis presents the most significant barrier to prompt treatment and such injuries should be suspected when a bile leak persists despite “normal” cholangiography, and there is a presumed failure of a “CD stump leak” closure after biliary stent placement.^2^ In addition, anatomical variation of the biliary tree is not uncommon and can increase the risk of ductal injuries.^3^ Surgeon awareness of the variant biliary branching is essential to decrease the risk of accidental damage during cholecystectomy and prevent its associated significant morbidity.^4^

While most bile leaks after cholecystectomy can be managed conservatively via ERCP and biliary stent placement, alternative treatment options must be explored when conservative managements fail. The case presentation in our patient and repeat ERCP were most consistent with leak from rare anatomic variations of the right posterior sectoral duct system, which denotes class V for individual right sectoral bile duct injury according to the Bismuth–Strasberg system for classifying iatrogenic bile duct injuries.^5^

Perera et al.^6^ proposed that nonoperative management is feasible in the majority of patients with leaks secondary to right posterior sectoral duct injuries via percutaneous drain placement and endoscopic stenting. In cases where the leak persists, operative intervention should be sought. Although hepatic abscess can be resulted with posterior sectoral bile duct ligation, it can be safely considered in small duct size as performed in our case.^6^ Partial hepatic resection or biliary reconstruction via Roux‐en‐Y hepaticojejunostomy might be required based on intraoperative assessment, biliary anatomy, underlying liver disease, and patient comorbidities.^7^

## 4 CONCLUSION

We conclude that subvesical bile duct injury may rarely present with delayed abdominal pain and fever after cholecystectomy. Avoiding deep dissection into liver parenchyma and staying close to the gallbladder can potentially avert injury to these ducts. Subvesical bile duct leaks that cannot be managed by endoscopic biliary stenting and external drains may need surgical intervention for definitive control of the bile leak.

## AUTHOR CONTRIBUTIONS

Drs. Sharma, Ruch, Alwatari, Bouhaidar, and Ms. Lele were responsible for manuscript design and data acquisition. All authors contributed to drafting the manuscript and revising it critically and provided approval for the final manuscript version.

## CONFLICT OF INTEREST

None.

### CONSENT

Written informed consent was obtained from the patient to publish this report in accordance with the journal's patient consent policy.

## Data Availability

None.
